# Experimental Study on Acoustic Emission Characteristics of Modified Phosphogypsum at Different Loading Rates

**DOI:** 10.3390/ma18112491

**Published:** 2025-05-26

**Authors:** Bo Zhang, Ji Zhang, Qiaoli Le, Duoduo Wang, Jiangtao Ding, Chaohua Xu

**Affiliations:** School of Civil Engineering and Architecture, Guizhou Minzu University, Guiyang 550025, China; zhangbo_dzs@126.com (B.Z.); jzhang2007@126.com (J.Z.);

**Keywords:** solid waste, modified phosphogypsum, loading rates, acoustic emission, mechanical properties

## Abstract

Modified phosphogypsum (MPG) is a new type of solid waste, which could show unique mechanical properties in complex stress conditions. In this study, the effects of different loading rates (0.05, 0.1, 0.5, and 1 MPa/s) on the mechanical properties and acoustic emission (AE) characteristics of modified phosphogypsum were systematically studied through uniaxial compression tests combined with AE technology. The results showed that (1) the peak strength and elastic modulus of MPG increased as a power function of the loading rate, while the peak strain gradually decreased. (2) The cumulative event count of AE decreased as a power function with an increasing loading rate. Compared to the lowest loading rate, the cumulative event count was reduced by nearly two orders of magnitude. (3) An increase in the loading rate resulted in greater large-scale macroscopic failure in MPG specimens, along with an increased proportion of low-frequency AE signals and tensile cracks. (4) The *b*-value of AE decreased with an increasing loading rate, suggesting that microcrack-dominated small-scale damage prevailed at low loading rates, whereas large-scale damage became more pronounced at high loading rates. The abrupt drop in the *b*-value served as a precursor signal for macroscopic failure. This study presents an innovative methodology combining variable loading rates with AE technology to investigate the mechanical response of MPG, and the findings reveal the influence of the loading rate on the mechanical properties and AE characteristics of MPG, providing a theoretical basis for its engineering application under different loading environments.

## 1. Introduction

Phosphogypsum is a waste product generated during the wet-process production of phosphoric acid, with its main component being calcium sulfate dihydrate (CaSO_4_·2H_2_O). According to incomplete statistics, the global annual output of phosphogypsum is approximately 280 million tons, and the accumulated stockpile has reached 7 billion tons [1]. The complex composition and high impurities of phosphogypsum lead to its low comprehensive utilization rate in various fields [2]. At present, the main treatment method is stockpiling [3]. However, the hazardous pollutants in the stored phosphogypsum may contaminate local air, soil, and water sources due to the effects of strong winds, rainfall, and seepage [4]. In this regard, researchers have used techniques such as decontamination, washing, roasting, flotation, and neutralization [5,6,7,8,9] to treat the raw PG in an environmentally sound manner before applying it in the fields of agriculture [10], chemical industry [11], and the environment [12]. However, phosphogypsum is consumed in limited quantities and at a high cost.

Scholars have modified phosphogypsum to make building materials such as cement retarders [13], gypsum boards [14], gypsum blocks [15], and mine fillers [16], which can consume large amounts of phosphogypsum and effectively reduce carbon emissions. Therefore, the physical and mechanical properties of modified phosphogypsum (MPG) have garnered increasing attention in recent studies. Xiao et al. [17] used the bioinduced carbonate precipitation method to determine the optimal formulation of curing stabilized MPG and investigated the physico-mechanical properties, such as particle gradation, the permeability coefficient, and the unconfined compressive strength of MPG with each material ratio. Wu et al. [18] employed the bioinduced carbonate precipitation method to identify the optimal formulation for curing-stabilized MPG and further examined its physico-mechanical properties, including particle gradation, thermogravimetric analysis results, and unconfined compressive strength, across various material ratios.

Acoustic emission (AE) technology is a non-destructive testing method that utilizes sensors to detect, record, and quantitatively analyze AE signal characteristics, providing insight into the precursor information of progressive failure in brittle materials such as concrete and rock [19]. Therefore, AE technology is of great significance for studying the damage evolution and fracture mechanism of engineering materials [20], which has been widely used in the field of civil engineering materials [21,22,23]. Zheng et al. [24] employed the rise amplitude (RA) and average frequency (AF) parameters derived from the acoustic emission (AE) technique to investigate the fracture mechanisms associated with various Type I fracture toughness testing methods and further elucidated the substantial differences in high-density regions of RA-AF distribution among specimens with varying geometries. Li et al. [25] carried out uniaxial compression tests on granite with different strain rates, and the *b*-value of AEs decreased with an increasing strain rate, i.e., low-amplitude AE activity dominated at low strain rates, whereas intense AE activity accounted for a larger proportion at high strain rates. Chen et al. [26] carried out three-point bending tests on concrete. The cumulative ringing count of AEs over time exhibited two distinct inflection points. The first inflection point corresponded to the peak load, and following this point, the concrete’s load-bearing capacity transitioned into a softening phase. Eid et al. [27] performed axial compression tests on high-strength concrete columns, and most of the AE events were located in the surface layer of the concrete, especially those corresponding to macroscopic cracks.

The above studies on the mechanical properties of MPG were carried out under a single loading rate. As a typical brittle material, the mechanical properties of MPG are significantly correlated to the loading rate. Therefore, it is particularly necessary to analyze the effect of the loading rate on mechanical properties by AE technology. In this study, AE technology is used to monitor uniaxial compression tests under various loading rates. This investigation aims to explore the mechanical properties and AE characteristics of MPG under different loading conditions, thereby providing a robust theoretical foundation for applying MPG across diverse engineering fields.

## 2. Materials and Methods

### 2.1. Materials

The principle of MPG involves utilizing calcium-based alkaline materials to neutralize acidic components and transform them into insoluble compounds, such as calcium phosphate. This process effectively minimizes the adverse effects of harmful impurities, thereby improving the performance and strength of MPG. Based on the treatment scheme of lime neutralization and modification of hemihydrate phosphogypsum by Wu et al. [18], this study prepared specimens using a mixture of hemihydrate phosphogypsum, lime, a melamine-based water reducer, and water. The specific proportioning scheme is presented in Table 1. Hemihydrate phosphogypsum appears off-white to gray, exists in a powder form, exhibits an acidic pH of 3.53, and contains 20% to 25% crystallization water. The mass fraction of available CaO in quicklime is not less than 70%. The water-reducing agent is the F10 melamine water-reducing agent.

**Figure 1:**
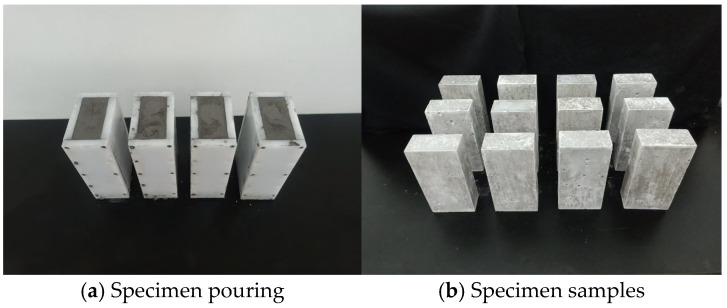
Preparation of MPG specimens.

The fabrication process of the specimen is as follows:

(1) The length, width, and height of the specimen casting mold are 75, 37.5, and 150 mm, respectively. The actual amount of each material required is calculated according to the material ratio scheme in Table 1 and weighed in a container.
(2) First, the semi-aqueous phosphogypsum and quicklime are thoroughly mixed. Subsequently, melamine water reducer and water are added while continuously stirring the thick mud mixture until homogeneity is achieved. The resulting mixture is then poured into the mold (Figure 1a).
(3) After disassembling the mold of the poured MPG specimen, the molded MPG test sample is obtained by curing it under natural conditions for 28 days (Figure 1b).

### 2.2. Theoretical Methods

When a material experiences deformation or failure, resulting in either microscopic or macroscopic damage (such as the initiation and propagation of cracks), elastic waves are emitted. As a non-destructive detection method, AE technology can capture the changes in elastic waves in this process.

**Figure 2:**
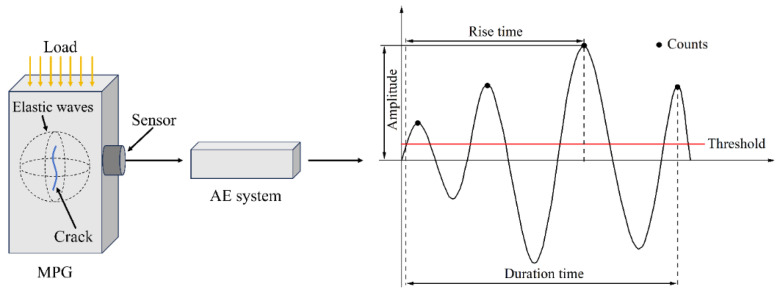
Schematic diagram of AE waveform and characteristic parameters.

Figure 2 illustrates the schematic diagram of the AE waveform and characteristic parameters. The AE-related feature parameters are explained as follows:

(1) Threshold: A specific value, usually set to reduce noise processing, can be monitored and recorded when the signal strength exceeds this value, in dB.
(2) Amplitude: The maximum amplitude value of the AE signal waveform, in dB.
(3) Event count: AE signals greater than the threshold are recorded as an AE event.
(4) Rise time: The time interval from the first time the AE signal exceeds the threshold value to the maximum amplitude, in μs.
(5) Duration: The time interval from when the AE signal is greater than the threshold value for the first time to when it is less than the threshold value, in μs.

The number of events reflects the frequency of AE activities inside the material, with a higher count of events corresponding to a higher degree of internal damage. The amplitude serves as a critical parameter for characterizing the strength of the AE signal and is commonly utilized as an indicator to assess the relative magnitude of AE events. A higher amplitude generally correlates with greater damage to the specimen. AE energy characteristics can reflect the energy release scale of AE events and are important parameters for evaluating the degree of internal damage to materials.

**Figure 3:**
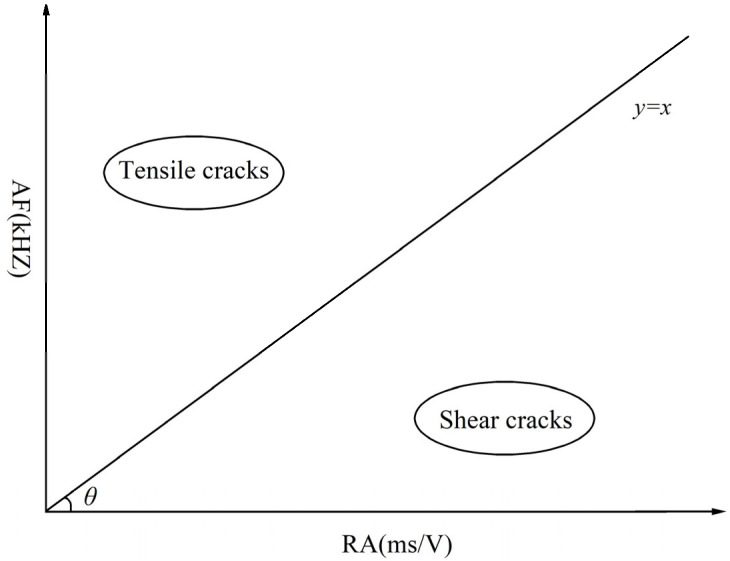
RA-AF value and crack type.

Among the characteristic parameters of the AE signal, RA is the ratio of the rise time to the amplitude, and AF is the ratio of the number of events to the duration, expressed by Equations (1) and (2). According to existing research [28,29,30], a larger AF value and a smaller RA value are typically associated with tension ruptures, whereas a smaller AF value and a larger RA value are generally linked to shear ruptures. The boundary line between tension cracks and shear cracks is defined by y = x. Therefore, when analyzing AE signal parameters, the distribution of RA values and AF values is often used to qualitatively analyze the damage type and the related quantity of cracks inside the rock. As shown in Figure 3, data points on the upper side of the y = x line represent tension failure, and data points on the lower side of the y = x line represent shear failure.

(1) $$\mathrm{RA}=\frac{\mathrm{Rise}\;\mathrm{time}}{\mathrm{Amplitude}}$$

(2) $$\mathrm{AF}=\frac{\mathrm{Counts}}{\mathrm{Duration}\;\mathrm{time}}$$

### 2.3. Test System

**Figure 4:**
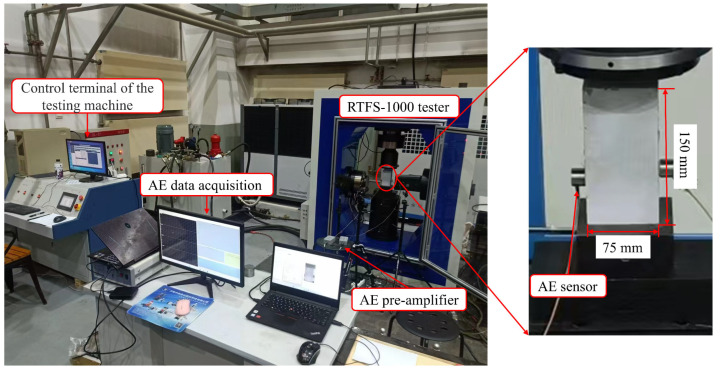
Test system.

The test system consists of a loading device and an AE acquisition device (Figure 4). The loading device is the RTFS-1000 (Huiyang Scientific Instrument Co., Ltd., Changchun, China) variable-frequency and variable-amplitude rock dynamic shear system. Equipped with a hydraulic control servo system, it can perform variable-frequency dynamic testing within the range of 0.01–10 Hz, provide a maximum axial test force of 1000 kN, and achieve a data sampling interval of 0.05 s. During the test, the loading device is capable of recording and monitoring axial deformation and axial load data in real time, as well as synchronously generating data curves. The AE acquisition device is a DS5-8A AE signal acquisition system (Softland Times Technology Co., Ltd., Beijing, China), which can simultaneously acquire data from up to 8 channels with a sampling frequency of 3 MHz. The sensor is an RS-24A sensor (Softland Times Technology Co., Ltd., Beijing, China) with a frequency range of 100 to 500 KHz. The AE signal generated during the loading process of the specimen is enhanced by a fixed-gain amplifier with a built-in bandpass filter, and the AE data are processed by relevant software.

### 2.4. Test Methods

In order to ensure that the end faces of the MPG specimens are flat and smooth and to enable axial compression during loading, the end faces must be polished before testing, ensuring that the flatness of both end faces is less than 0.02 mm. The length, width, height, and mass of the MPG specimens were measured using a vernier caliper and an electronic scale, and the density of each specimen was calculated. The AE sensors were placed on both sides of the specimen and fixed with a coupling agent evenly applied between the specimens (Figure 4). To effectively filter out mechanical and human-induced noise, the threshold was set at 40 dB. Any signal exceeding this threshold was classified as an AE event. A total of 12 MPG specimens with dimensions of 75 mm × 37.5 mm × 150 mm were prepared for this experiment. The loading procedure was load-controlled with prescribed rates of 0.05, 0.1, 0.5, and 1 MPa/s, and three specimens were tested under each rate. The loading device and the AE acquisition device were synchronized to start and end simultaneously. If the specimen exhibited macroscopic cracking or if there was a sudden drop in the stress–strain curve during loading, the test was terminated.

## 3. Results and Discussion

### 3.1. Characteristics of Damage and Mechanical Properties

#### 3.1.1. Destruction Characteristics

**Figure 5:**
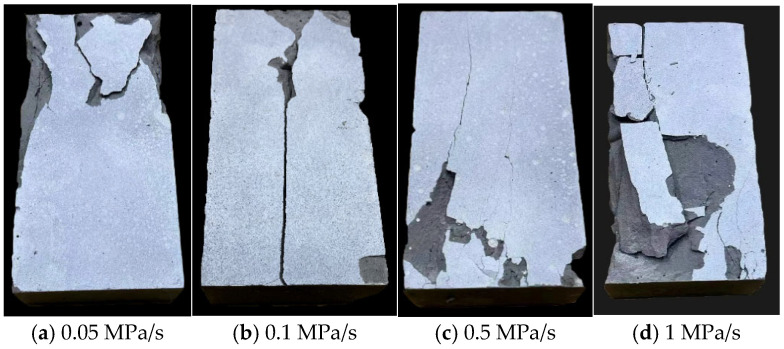
Macroscopic failure characteristics of MPG specimens at different loading rates.

The material used in this test, namely the MPG, is a typical brittle material. Consequently, none of the specimens exhibited clear fracture characteristics during loading until macroscopic cracks appeared instantaneously upon reaching the peak strength. Figure 5 illustrates the failure modes of the MPG specimens after being loaded at various loading rates. At a loading rate of 0.05 MPa/s, fragments of the specimen spall off, and no distinct through-fracture surface is observed. When the loading rate reaches 0.1 MPa/s, the specimen starts to exhibit a through-thickness fracture surface. When the loading rate exceeds 0.5 MPa/s, the spalling phenomenon in the MPG specimen becomes more pronounced, and the fracture condition deteriorates significantly.

#### 3.1.2. Mechanical Properties

**Figure 6:**
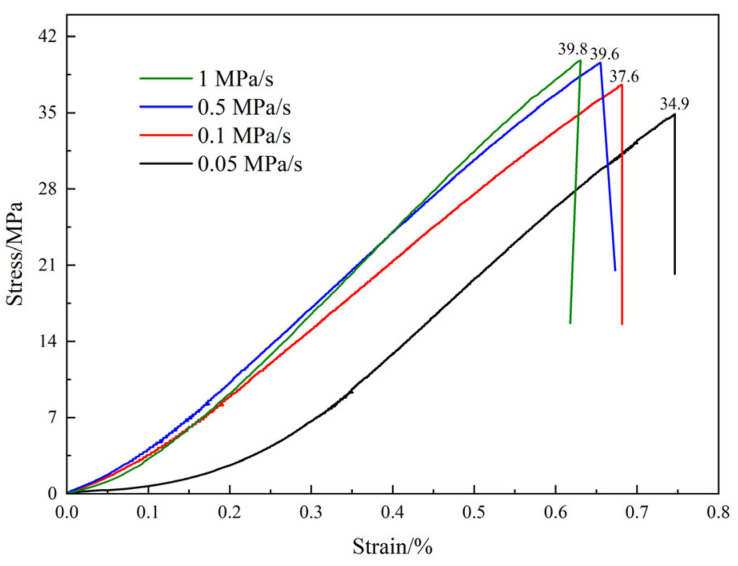
MPG stress–strain curves at different loading rates.

**Figure 7:**
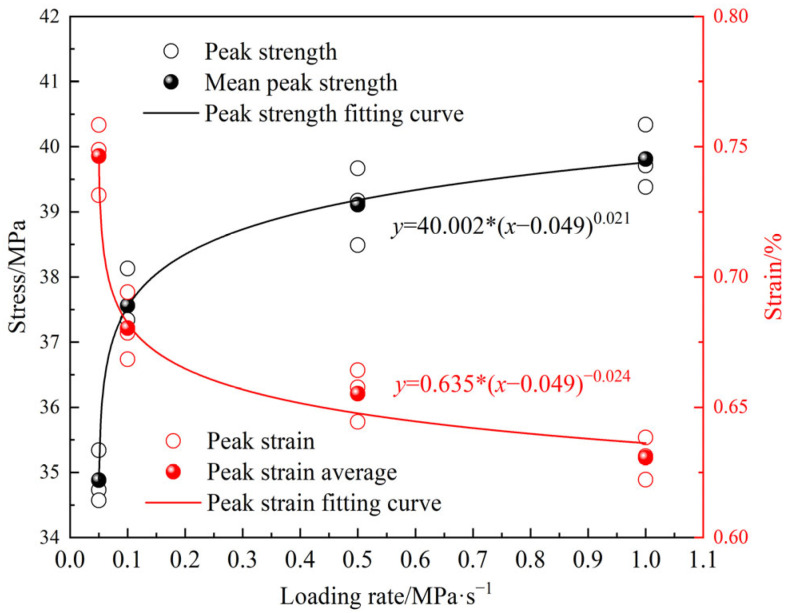
Relationship between peak strength, peak strain, and loading rate.

**Figure 8:**
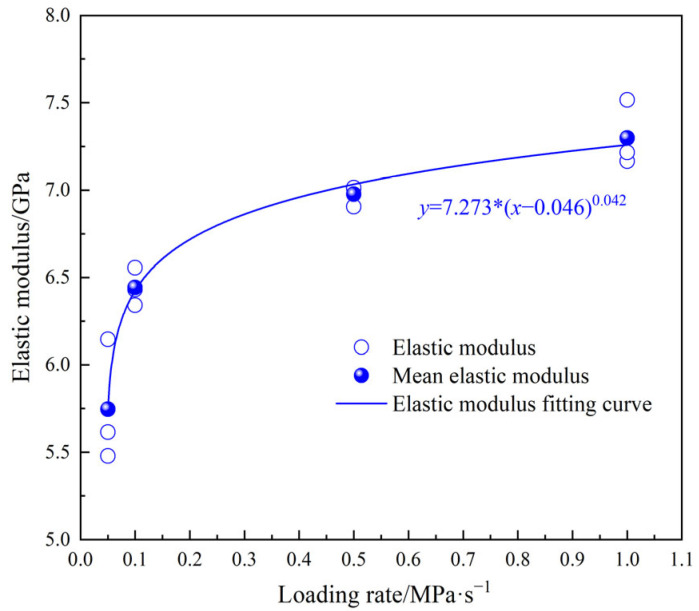
Relationship between elastic modulus and loading rate.

Figure 6 presents the stress–strain curves of MPG under various loading rates. At each loading rate, the post-peak stage of the MPG specimen curve exhibits a sharp decline, suggesting that the mechanical behavior of MPG resembles that of typical brittle materials, such as rock and concrete. The mechanical properties of MPG show a significant sensitivity to the loading rate. That is, the peak strength and slope of the curve increase with the increase in the loading rate, and the peak strain decreases with the increase in the loading rate. This is because the mechanical properties of the MPG specimens are influenced by the friction and slip behavior of the cementitious substances within the material. A lower loading rate leads to a prolonged loading duration before specimen failure. Additionally, the initial stress–strain curve exhibits a relatively gentle slope as a result of the adequate compaction of the specimen. As the stress continues to increase, the internal damage and cracks of the specimen are fully developed, resulting in lower peak strength, elastic modulus, and larger peak strain. Under higher loading rates, the stress–strain curve exhibits a shorter crack compaction stage. Consequently, the internal friction and slip processes within the MPG material conclude more rapidly, leading to less fully developed microcracks and damage within the specimen. Additionally, the MPG specimen lacks sufficient time to release the accumulated energy. Large-scale fractures developed in the specimen as it approached the failure stage, exhibiting pronounced brittle failure characteristics. This resulted in a higher peak strength and elastic modulus but a lower peak strain. The peak strength, peak strain, and elastic modulus have a power function relationship with the loading rate. When the loading rate is greater than 0.1 MPa/s, the influence of the loading rate on the above mechanical properties gradually decreases (Figure 7 and Figure 8).

### 3.2. AE Characteristics

#### 3.2.1. Cumulative Event Count, Amplitude, and Energy Characteristics of AE

**Figure 9:**
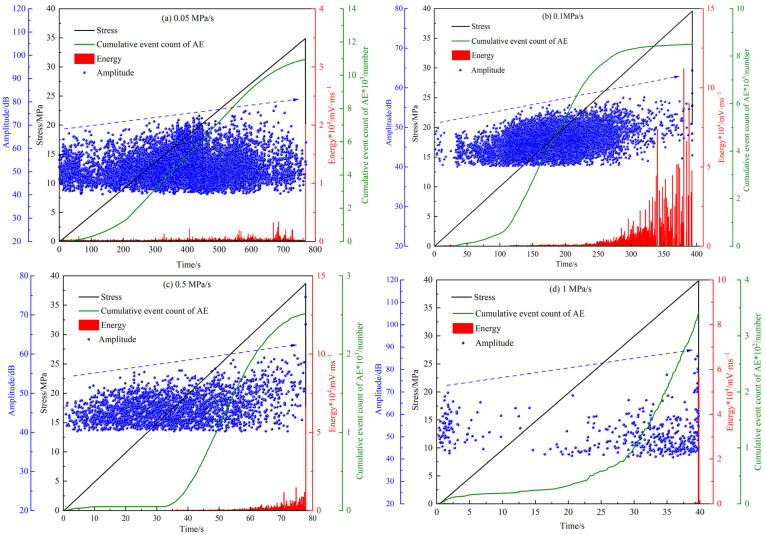
Relationship between AE impact count, amplitude, energy, and loading time at different loading rates.

As illustrated in Figure 9, no significant damage is observed in the specimen during the compaction and elastic loading stages. Consequently, both the cumulative count of AE events and the energy rate remain low and exhibit steady growth. After entering the crack propagation stage, many new microcracks emerge inside the specimen, and these microcracks continuously expand and interconnect with each other, forming macrocracks, with the cumulative count of AE events and energy rate increasing sharply. When the specimen approaches failure, it loses its load-bearing capacity, and both the cumulative count of AE events and the energy rate reach their peak values. During the entire loading process, the amplitude gradually increases with the increase in stress (blue dashed line).

**Figure 10:**
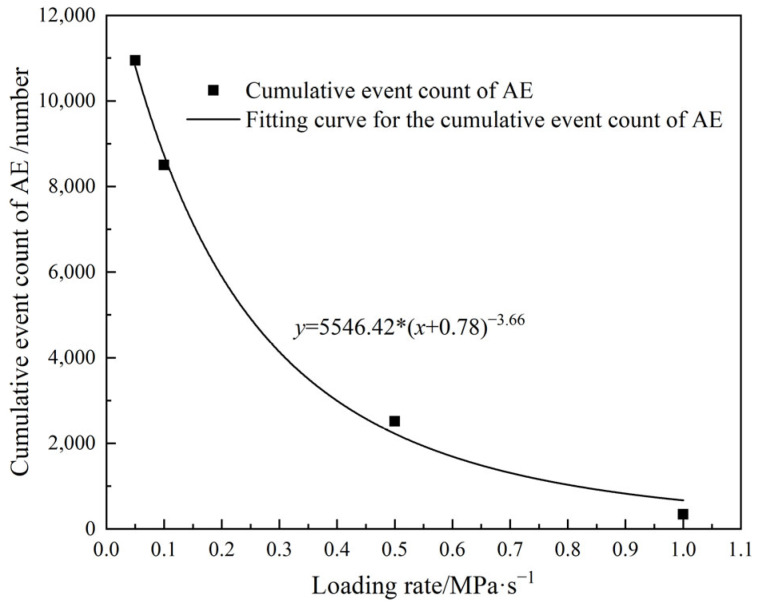
Relationship between the cumulative count of AE events and loading rate.

However, the cumulative count of AE events differs significantly at different loading rates. As the loading rate increases, the maximum cumulative count of AE events gradually decreases, and the cumulative count of AE events and the loading rate are in a power function relationship (Figure 10). Compared with the loading rate of 0.05 MPa/s, the corresponding cumulative count of AE events is reduced by almost two orders of magnitude. At a low loading rate, the particles within the MPG specimen have sufficient time to undergo rubbing and slipping, resulting in complete damage and the generation of a large number of AE signals. When the loading rate is high, the specimen is not fully damaged, and the cracks do not have enough time to develop, leading to fewer AE signals.

#### 3.2.2. Frequency Characteristics

**Figure 11:**
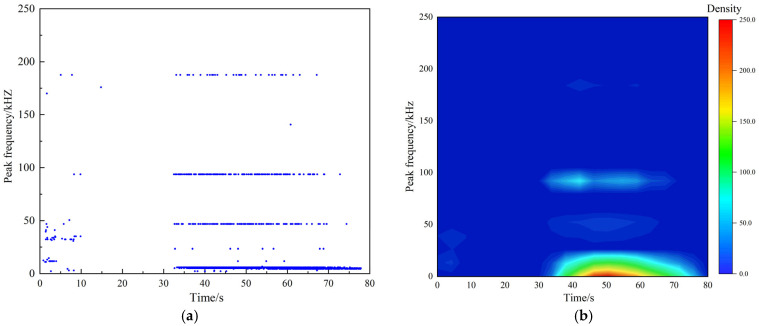
Distribution of peak frequency. (**a**) Relationship between peak frequency distribution and time; (**b**) Peak frequency density cloud diagram.

AE peak frequency is an important parameter to describe the characteristics of AE signals. It refers to the frequency point with the largest amplitude in the signal spectrum, also known as the center frequency or main frequency of the signal. Low-frequency signals are usually associated with large-scale or macroscopic rupture events, which may originate from large-scale crack extension or sliding of the fracture surface. High-frequency signals are usually associated with small-scale or microscopic fracture events, such as the formation and propagation of microcracks. These signals are generally related to local stress concentration or rapid deformation of small areas within the material [31]. Taking the test at a loading rate of 0.5 MPa/s as an example (Figure 11), the peak frequencies of the AE signals generated during the loading process are all distributed below 300 kHz, primarily consisting of low-frequency signals and exhibiting a band-like distribution pattern. Similar peak frequency distributions can also be observed in other loading rate tests. Therefore, 0–50 kHz is defined as a low-frequency signal, 50–100 kHz is defined as a medium-frequency signal, and 100 kHz and above is defined as a high-frequency signal.

**Figure 12:**
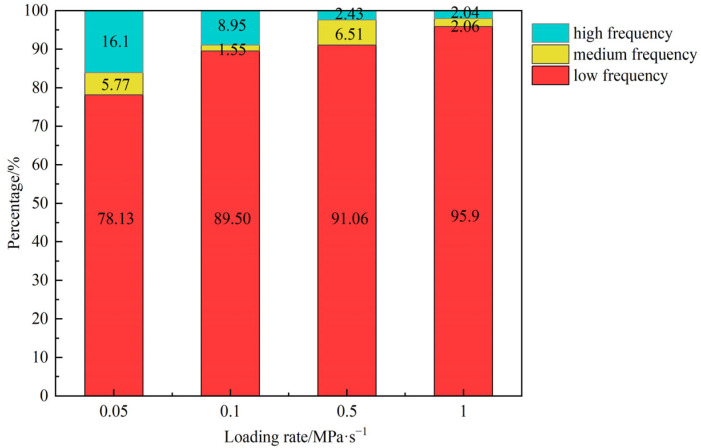
Distribution of the main frequency of AE signal at different loading rates.

In order to analyze the relationship between the peak frequency of the AE signal and the loading rate more specifically, Figure 12 plots the distribution percentages of the three peak frequency bands (0–50 kHZ, 50–100 kHZ, 100 kHZ and above) at different strain rates. As the loading rate increases, the proportion of low-frequency signals (0–50 kHz) gradually increases, while the proportion of high-frequency signals (100 kHz and above) decreases. This is because the lower loading rate provides enough time for microcracks to fully initiate and expand, thus generating a large number of high-frequency signals. A higher loading rate will produce larger macrocracks, resulting in a gradual increase in the proportion of low-frequency signals. In addition, owing to the dispersive characteristics of the material, the high-frequency components of the AE signal exhibit faster decay in the time domain, making them less likely to be detected by the sensor.

#### 3.2.3. Distribution Characteristics of RA-AF Values

**Figure 13:**
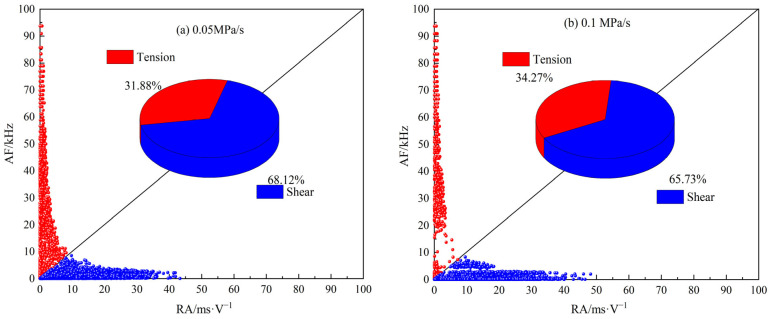

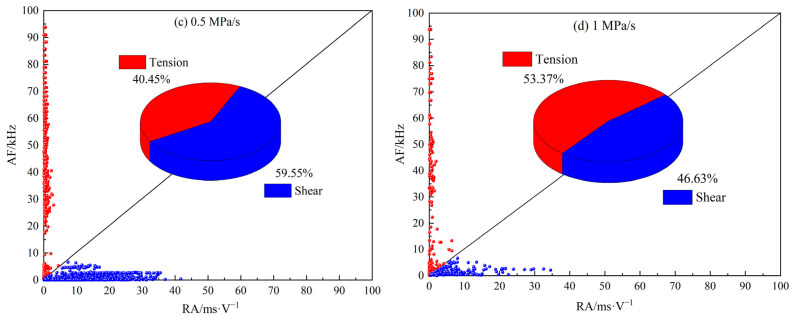
Distribution and proportion of RA-AF values at different loading rates.

As shown in Figure 13, under the action of a low loading rate, the RA-AF value is mainly distributed in the horizontal coordinate, and the proportion of shear cracks is high. With the increase in loading rate, the proportion of tension cracks gradually increases. This indicates that the increase in loading rate changes the rupture mechanism of MPG to some extent, which is in general agreement with the relevant study on rocks in reference [32]. It should be noted that the absolute number of tension cracks and shear cracks distinguished by the RA-AF value method lacks precision. However, this method effectively reflects the relative changes in the proportion of tension versus shear cracks.

#### 3.2.4. *b*-Value Characteristics

In seismology, Gutenberg–Richter established the G-R criterion for the statistical relationship between magnitude and frequency, and the *b*-value is derived from the parameters of the G-R criterion [33], as expressed by Equation (3).

(3) lgN=a−bM

where *N* is the number of earthquakes with a magnitude greater than *M*; *M* is the magnitude; *a* and *b* are constants.

Since the AE elastic waves and seismic waves generated during the material damage process are similar in principle, the magnitude is substituted with the amplitude when calculating the AE *b*-value. The AE *b*-value is then obtained through linear fitting using the least squares method described in Equation (4).

(4) $$\mathrm{lg}N=a-b(A_{\mathrm{dB}}/20)$$

where *N* is the number of AE events; *A*_dB_ is the AE amplitude; *a* and *b* are the least squares linear fitting parameters, and parameter *b* is the slope of the linear fitting line, which is the desired AE *b*-value.

**Figure 14:**
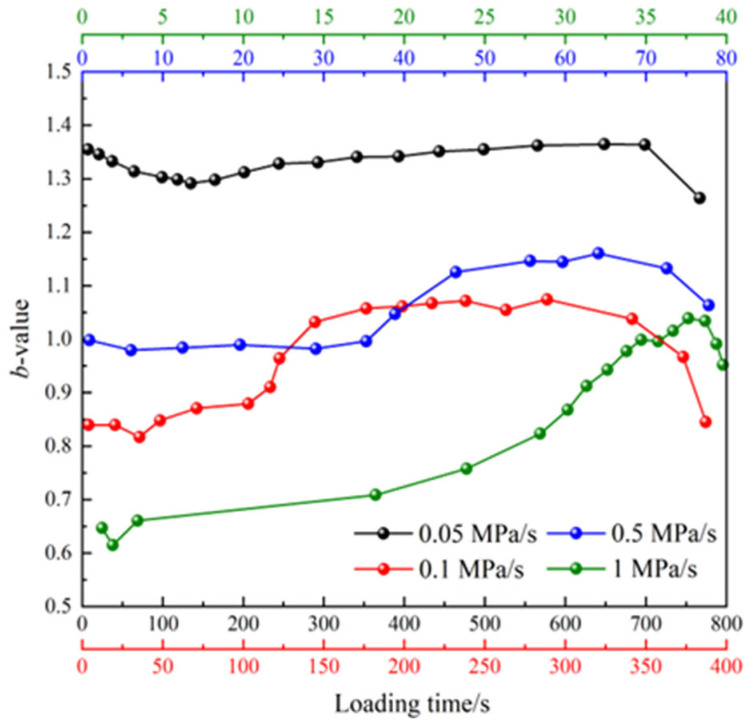
Distribution of main frequency of condition signal at different loading rates.

The *b*-value can serve as an indicator to reflect the proportion of the magnitude-scale distribution of AE events, effectively characterizing the evolution of internal damage during material compressive failure. Moreover, it provides a reliable method for analyzing and identifying precursor information related to material rupture. Therefore, this study calculates the *b*-value within the loading time to effectively reflect the crack evolution characteristics of the MPG specimen during the loading process. The AE amplitude data are counted in intervals of 5 dB, as the cumulative event count of AE for MPG specimens decreases gradually with increasing loading rates. To clearly compare the changes in the *b*-value curve, an appropriate AE event step size is selected under varying loading rates for *b*-value calculation. Subsequently, the least squares method described in Equation (4) is employed to fit and calculate the *b*-value. The results are presented in Figure 14.

It can be observed from the figure that the evolution process of the *b*-value under different loading rates exhibits a similar trend, characterized by a gradual increase at the onset of loading. This suggests that the initiation of microcracks remains the dominant factor in the specimen’s damage process. When approaching failure, the *b*-value decreases, indicating that the sample has entered the macrocrack propagation stage. The evolution of the *b*-value at a loading rate of 0.05 MPa/s is a bit special. It first undergoes a decreasing process, indicating more low-amplitude AE events. For loading rates of 0.5 MPa/s and 1 MPa/s, the *b*-value drops sharply in the later stage of loading, which is usually considered a precursor of macroscopic failure. In addition, the range of *b*-values exhibits significant variation under different loading rates. Typically, the *b*-value decreases as the loading rate increases, suggesting that small-scale damage predominates at low loading rates, whereas severe damage becomes more prominent at high loading rates.

## 4. Conclusions

In this study, AE technology was used as a monitoring method to conduct uniaxial compression tests on MPG at different loading rates. The effects of loading rates on the mechanical properties and AE characteristics of MPG were systematically analyzed. The main conclusions are as follows:

(1) MPG is a typical brittle material, and its mechanical properties are sensitive to loading rate. The peak strength and elastic modulus increase with the increase in loading rate, while the peak strain decreases with the increase in loading rate. When the loading rate is greater than 0.1 MPa/s, the influence of the loading rate on the above mechanical properties gradually decreases.
(2) As the loading rate increases, the maximum cumulative count of AE events gradually decreases, indicating an inverse relationship between the two. Furthermore, the cumulative count of AE events and the loading rate follow a power function relationship. Compared with the lowest loading rate, the cumulative count of AE events at specimen failure was reduced by almost two orders of magnitude at the highest loading rate.
(3) With the increase in loading rate, large-scale macroscopic damage becomes increasingly significant, leading to a gradual increase in the proportion of low-frequency AE signals. In addition, the rupture mechanism of MPG has also changed to a certain extent, and the distribution of RA-AF data points shows that the proportion of tensile cracks has gradually increased.
(4) The *b*-values show considerable variation across different loading rates. Generally, the *b*-value decreases with an increase in the loading rate, indicating that small-scale damage is more prevalent at lower loading rates, while more severe damage tends to occur at higher loading rates. For loading rates of 0.5 MPa/s and 1 MPa/s, the *b*-value drops sharply in the later stage of loading, which is usually considered a precursor of macroscopic failure.

## Figures and Tables

**Table 1:** Material ratio of MPG specimens.

Material Composition	Hemihydrate Phosphogypsum	Quicklime	Melamine Water-Reducing Agent	Water
Percentage composition	97%	3%	1%	29.1%

## Data Availability

The original contributions presented in this study are included in the article. Further inquiries can be directed to the corresponding author.
